# Redox mechanisms and their pathological role in prion diseases: The road to ruin

**DOI:** 10.1371/journal.ppat.1011309

**Published:** 2023-04-27

**Authors:** Jereme G. Spiers, Hsiao-Jou Cortina Chen, Joern R. Steinert

**Affiliations:** 1 Clear Vision Research, Eccles Institute of Neuroscience, John Curtin School of Medical Research, College of Health and Medicine, The Australian National University, Acton, Australian Capitol Territory, Australia; 2 School of Medicine and Psychology, College of Health and Medicine, The Australian National University, Acton, Australian Capitol Territory, Australia; 3 The Department of Biochemistry and Chemistry, La Trobe Institute for Molecular Science, La Trobe University, Bundoora, Victoria, Australia; 4 Metabolic Research Laboratories, Wellcome Trust MRC Institute of Metabolic Science, University of Cambridge, Addenbrooke’s Hospital, Cambridge, United Kigdom; 5 Division of Physiology, Pharmacology and Neuroscience, University of Nottingham, School of Life Sciences, Nottingham, United Kigdom; National Institutes of Health, UNITED STATES

## Abstract

Prion diseases, also known as transmissible spongiform encephalopathies, are rare, progressive, and fatal neurodegenerative disorders, which are caused by the accumulation of the misfolded cellular prion protein (PrP^C^). The resulting cytotoxic prion species, referred to as the scrapie prion isoform (PrP^Sc^), assemble in aggregates and interfere with neuronal pathways, ultimately rendering neurons dysfunctional. As the prion protein physiologically interacts with redox-active metals, an altered redox balance within the cell can impact these interactions, which may lead to and facilitate further misfolding and aggregation. The initiation of misfolding and the aggregation processes will, in turn, induce microglial activation and neuroinflammation, which leads to an imbalance in cellular redox homeostasis and enhanced redox stress. Potential approaches for therapeutics target redox signalling, and this review illustrates the pathways involved in the above processes.

## Introduction

Prion disease or transmissible spongiform encephalopathies are a type of rare, progressive, and fatal neurodegenerative disorders caused by misfolding of the cellular prion protein (PrP^C^) and accumulation of its disease-associated scrapie prion isoform (PrP^Sc^). Symptoms of prion disease include rapidly developed dementia, difficulty thinking and speaking, impaired coordination, and behavioural changes. There is no known cure for prion disease, with current treatment focused primarily on symptom management. The exact molecular mechanisms of prion disease are yet to be fully characterised, but hallmark histopathological features can be observed including the development of widespread spongiform lesions, neuronal loss, and gliosis. Recently, there has been a great deal of interest in the role of redox imbalance/oxidative stress in contributing to those aforementioned clinical features of prion disease. This heavily coincides with disruption of metal homeostasis in the brain, particularly redox-active metals such as iron and copper, which may further contribute to the associated oxidative stress by acting as an important neurotoxicity trigger. Here, we summarise the advances in understanding the functional link between redox stress and prion disease.

### Prion and redox imbalance/oxidative stress

Neuroinflammatory redox stress is a common observation in the pathophysiological development and progression of prion diseases in both animal models and human forms of the disease [1–3]. This neurodegenerative neuroinflammation generally involves microglial cells, with the polarisation of these towards an “activated” M1 phenotype resulting in the release of pro-inflammatory cytokines and an up-regulation of oxidative/nitrosative stress machinery to combat potential pathogens [4]. While microglial-derived neuroinflammation is generally viewed as a mechanism leading to neuronal death, particularly where excessive oxidative stress occurs, in prion disease, this mechanism becomes less clear-cut. Microglia become classically activated at the site of spongiform lesions and display this phenotype in the early stages of infection prior to neuronal death, suggesting they play a role in prion-induced neurodegeneration [5]. Activated microglia express high levels of the superoxide radical-generating NADPH oxidase enzyme capable of inducing oxidative stress in neighbouring cells, which may contribute to the neurodegenerative phenotype seen in prion disease. Moreover, the antioxidant superoxide dismutase (SOD) enzymes also show reduced expression in the early development of prion disease, which likely exacerbates NADPH-oxidase-derived oxidative stress [6].

Importantly, microglial proliferation and activation also appears to be important in the effective clearance of prions, with pharmacological-mediated reductions in microglia resulting in enhanced prion deposition and neurotoxicity [7]. Moreover, the cytokine profile following prion infection is highly dependent on the prion strain and infected species, with some anti-inflammatory cytokines becoming more abundant in different rodent models and human Creutzfeldt–Jakob disease (CJD) [8,9]. Although up-regulated respiratory burst enzymes such as NADPH oxidase found in activated microglia play a critical role in prion-induced oxidative stress, mitochondrial dysfunction is also a key component of prion disease redox dysfunction. Mitochondrial oxidation is observed very early in prion disease pathogenesis and contributes to early oxidative stress [10–12]. A number of studies using different models of prion disease have shown the majority of differentially expressed proteins following infection mediate energy metabolism. This includes oxidative phosphorylation proteins such as ATP synthase, tricarboxylic acid cycle proteins including succinate dehydrogenase, aconitase 2, and malate dehydrogenase, and mitochondrial membrane proteins like mitoflin [13–16]. This broad degree of mitochondrial dysfunction contributes heavily to redox dysfunction and, together with pro-inflammatory microglial activation and reductions in primary antioxidant protection from SOD, likely forms the basis of oxidative stress in prion disease.

### Prion and nitrosative stress

The elevation of nitric oxide (NO) within the central nervous system is known to be associated with the pathogenesis of several neurodegenerative diseases including prion infection [17–19]. Using lipopolysaccharide to generate an inflammatory up-regulation of inducible nitric oxide synthase (iNOS) or treating cells with an NO-donor (sodium nitroprusside) significantly elevates both mRNA and protein expression of the prion protein via the MEK and p38 MAPK signalling pathways [20]. Up-regulation of this inflammatory NO-producing enzyme, iNOS, has also been demonstrated in brains of scrapie-infected mice, which may contribute to the observed vacuolation and astrocytosis [21,22]. Interestingly, in addition to the immune-related iNOS, constitutive endothelial NOS was also markedly increased in the hippocampus of ME7 scrappie-infected mice, which showed a degree of specificity in reactive astroglial cells and accumulated in the mitochondrial fraction [23]. The increase in NO and damaged mitochondria could, in turn, affect the function of the affected astrocytes and further contribute to the prion disease progression. In agreement with these studies, increased neuroinflammatory and altered nitric oxide signalling with an accompanied nitrergic stress have been observed in the hippocampus of hemizygous Tg37 mice that overexpress the cellular mouse prion protein using an alternative prion protein strain from Rocky Mountain Laboratory (RML) [24,25]. Suppression of NO production pharmacologically showed beneficial effects on hippocampal physiology and supressed prion protein misfolding highlighted the importance of nitrergic stress in the neuropathology of prion disease [24].

### Prion and biometals

Normal PrP^C^ is a well-known divalent metal binding protein with a high binding preference for copper ions in the N-terminal octa-repeat domain of the protein [26]. Moreover, the reactive oxygen species produced by Fenton chemistry have been shown to actively participate with redox-active divalent metals like copper to facilitate β-cleavage into N2 and C2 prion protein fragments [27–29]. The binding of copper appears to be highly important for PrP^C^ physiological functionality, with studies showing normal PrP^C^ possesses SOD1-like antioxidant activity, stimulates PrP^C^ endocytosis and trafficking activity, and modulates N-methyl-D-aspartate (NMDA) receptor activity [30–32]. Specifically, it has been shown that PrP^C^ and copper cooperatively modulate NMDA receptor activity by mediating S-nitrosylation to prevent neurotoxicity [33]. The N-terminal copper binding site of PrP^C^ also appears to be essential for neurite outgrowth, acting as a neurotrophic factor [34]. It is highly likely that the combination of these effects contributes to the neuroprotective actions of cellular PrP^C^. In prion-infected mice, it has been demonstrated that the reduction of NMDA receptor S-nitrosylation precedes the appearance of the clinical signs and neuropathological changes [35]. Therefore, dysregulation of NMDA receptor S-nitrosylation may act as a possible mechanism of neuronal death in prion pathology. During prion disease, the conversion of PrP^C^ to PrP^Sc^ is highly influenced by both the surrounding concentrations of available copper and the levels of copper bound to the PrP protein, with apo forms of the protein being more susceptible to conversion [36]. Moreover, the interaction of PrP^C^ with other redox-active divalent metals such as iron has enormous implications for the microenvironment surrounding PrP^Sc^ lesion sites. It has been shown previously that PrP^C^-null mice have impaired copper metabolism and the oxidase activity of copper-dependent ceruloplasmin, which is known to regulate iron mobilisation and distribution [37]. Accumulation and dysregulated iron release, in particular, can be highly deleterious to surrounding protein, lipids, and nucleic acids due to the high levels of oxidative stress produced via Fenton chemistry.

In addition to this direct involvement of biometals on local oxidative stress conditions, alterations in divalent metal homeostasis can influence gene expression via metal response elements in promotor regions of several genes [38,39] or indirectly via iron–sulphur (Fe–S) cluster containing proteins [40]. Metal dyshomeostasis can alter gene expression very early in the pathogenesis of prion disease before functional neuronal changes can be detected. Importantly, this can alter metal transporters and binding proteins capable of mediating cellular metal ion pools required for structural and functional incorporation into enzymes and chaperones. For example, divalent metal transporters Slc11a2 and Slc39a14 show reduced expression prior to symptomatic onset in prion diseased mice [6]. This is observed alongside a >50% reduction in expression of Ccs, the copper chaperone to SOD, responsible for redox-active copper insertion and proper protein folding of SOD1. Importantly, this suggests that SOD expression is not only reduced, but antioxidant activity is also compromised very early in prion disease pathogenesis.

### Outlook and treatment

**Fig 1:**
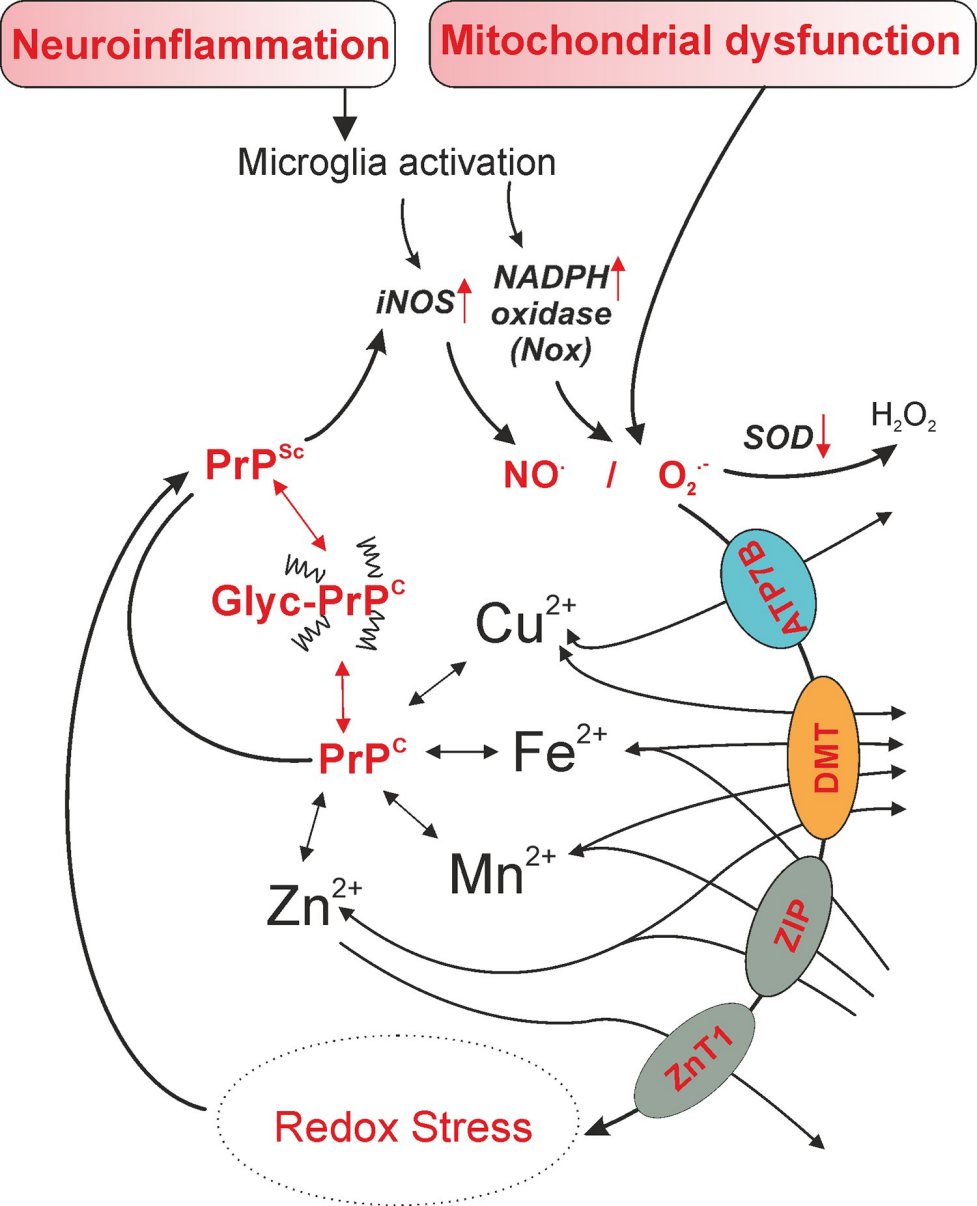
Hallmarks of prion disease pathogenesis include neuroinflammation and mitochondrial dysfunction, which facilitate production of reactive oxygen and nitrogen species via inflammatory-related enzymes including NADPH-oxidase (Nox) and inducible nitric oxide synthase (iNOS). These produce radical species such as superoxide (O_2_^**.-**^) and nitric oxide (NO^.^), respectively. Under normal conditions, antioxidants such as superoxide dismutase (SOD) would neutralise these radicals to less reactive species or reduce them to water. However, in pathological conditions such as prion disease where oxidant production is favoured, antioxidants are overwhelmed, which results in cellular redox stress. This is further exacerbated by the mobilisation of redox-active and transition metals such as iron (Fe^2+^), zinc (Zn^2+^), copper (Cu^2+^), and manganese (Mn^2+^), which causes dyshomeostasis in cellular biometal distribution through various divalent metal transporters (ZnT1, ZIP, DMT, ATP7B) and promotes oxidative stress. Together, these conditions may favour glycosylation of the native prion protein (PrP^C^), which facilitates conversion to the disease isoform (PrP^Sc^), further promoting disease progression.

Targeting biometals, such as copper and zinc, has been suggested as a potential therapeutic strategy for prion diseases. These biometals can bind to misfolded PrP^Sc^ proteins, which can inhibit protein aggregation. Biometals can also modulate the activity of certain enzymes involved in disease processes, ultimately slowing disease progression. In addition, an interaction between PrP^C^ and manganese or zinc, but not copper, causes shedding of the N1 fragment of PrP^C^, which, in turn, impacts prion N-glycosylation and its cellular functions, distribution, cellular trafficking, aggregation, and fibril formation [41,42]. In the unglycosylated mutants, PrP^C^ was localized on the cell surface and readily converted to PrP^Sc^, indicating that glycans are not necessary for prion infection but rather a lack of glycation favours misfolding and PrP^Sc^ transmission [43]. Thus, targeting of redox metal homeostasis will strongly impact prion function and transmissibility (Fig 1).

However, a main question of how to minimise or slow down prion propagation during early infection stages remains to be addressed. Early detection methods in animal models involve the use of aptamers [44], single-stranded nucleic acids (DNA, RNA) that are usually 22 to 100 nucleobases long and have molecular recognition properties similar to antibodies. Aptamers have also been investigated as novel anti-prion compounds. It was shown that peptide aptamers binding to PrP^C^ prion protein can abrogate prion propagation [45]. Binding of the designed anti-prion aptamer with PrP^C^ prion protein increased α-cleavage, interfered with protein internalization, and inhibited prion replication. A recent study also explored the possibility of reducing pro-inflammatory signalling of prion-infection in cell culture. This study provides some novel therapeutic insights by highlighting the ability of mesenchymal stromal cells in regulating inflammation by secreting anti-inflammatory small molecules to promote angiogenesis and neurogenesis [46].

In summary, the detection of early biomarkers and biosensors [47], identifying early stage infection, prion seeding and propagation [44], targeting of inflammatory processes, and biometal dyshomeostasis are essential steps to develop potential therapeutic strategies for prion diseases. However, more research is needed to determine the translational potential from animal models to humans.

## References

[1] Carroll JA, Striebel JF, Race B, Phillips K, Chesebro B. Prion infection of mouse brain reveals multiple new upregulated genes involved in neuroinflammation or signal transduction. J Virol. 2015;89(4):2388–2404. Epub 20141210. doi: 10.1128/JVI.02952-14 ; PubMed Central PMCID: PMC4338885. 25505076 PMC4338885

[2] Milhavet O, Lehmann S. Oxidative stress and the prion protein in transmissible spongiform encephalopathies. Brain Res Brain Res Rev. 2002;38(3):328–339. doi: 10.1016/s0165-0173(01)00150-3 . 11890980

[3] Milhavet O, McMahon HE, Rachidi W, Nishida N, Katamine S, Mange A, et al. Prion infection impairs the cellular response to oxidative stress. Proc Natl Acad Sci U S A. 2000;97(25):13937–13942. doi: 10.1073/pnas.250289197 ; PubMed Central PMCID: PMC17679. 11095725 PMC17679

[4] Burwinkel M, Riemer C, Schwarz A, Schultz J, Neidhold S, Bamme T, et al. Role of cytokines and chemokines in prion infections of the central nervous system. Int J Dev Neurosci. 2004;22(7):497–505. doi: 10.1016/j.ijdevneu.2004.07.017 . 15465279

[5] Betmouni S, Perry VH, Gordon JL. Evidence for an early inflammatory response in the central nervous system of mice with scrapie. Neuroscience. 1996;74(1):1–5. doi: 10.1016/0306-4522(96)00212-6 . 8843071

[6] Spiers JG, Chen HJC, Barry TL, Bourgognon JM, Steinert JR. Redox stress and metal dys-homeostasis appear as hallmarks of early prion disease pathogenesis in mice. Free Radic Biol Med. 2022;192:182–190. Epub 20220925. doi: 10.1016/j.freeradbiomed.2022.09.025 . 36170956

[7] Zhu C, Herrmann US, Falsig J, Abakumova I, Nuvolone M, Schwarz P, et al. A neuroprotective role for microglia in prion diseases. J Exp Med. 2016;213(6):1047–1059. Epub 20160516. doi: 10.1084/jem.20151000 ; PubMed Central PMCID: PMC4886355. 27185853 PMC4886355

[8] Baker CA, Lu ZY, Zaitsev I, Manuelidis L. Microglial activation varies in different models of Creutzfeldt-Jakob disease. J Virol. 1999;73(6):5089–5097. doi: 10.1128/JVI.73.6.5089-5097.1999 ; PubMed Central PMCID: PMC112554. 10233972 PMC112554

[9] Stoeck K, Bodemer M, Ciesielczyk B, Meissner B, Bartl M, Heinemann U, et al. Interleukin 4 and interleukin 10 levels are elevated in the cerebrospinal fluid of patients with Creutzfeldt-Jakob disease. Arch Neurol. 2005;62(10):1591–1594. doi: 10.1001/archneur.62.10.1591 . 16216944

[10] Choi SI, Ju WK, Choi EK, Kim J, Lea HZ, Carp RI, et al. Mitochondrial dysfunction induced by oxidative stress in the brains of hamsters infected with the 263 K scrapie agent. Acta Neuropathol. 1998;96(3):279–286. doi: 10.1007/s004010050895 . 9754961

[11] Keller G, Binyamin O, Frid K, Saada A, Gabizon R. Mitochondrial dysfunction in preclinical genetic prion disease: A target for preventive treatment? Neurobiol Dis. 2019;124:57–66. Epub 20181110. doi: 10.1016/j.nbd.2018.11.003 . 30423473

[12] Zhu T, Chen JL, Wang Q, Shao W, Qi B. Modulation of mitochondrial dynamics in neurodegenerative diseases: An insight into prion diseases. Front Aging Neurosci. 2018;10:336. Epub 20181105. doi: 10.3389/fnagi.2018.00336 ; PubMed Central PMCID: PMC6230661. 30455640 PMC6230661

[13] Kim MJ, Kim HJ, Jang B, Kim HJ, Mostafa MN, Park SJ, et al. Impairment of neuronal mitochondrial quality control in prion-induced neurodegeneration. Cell. 2022;11(17). Epub 20220902. doi: 10.3390/cells11172744 ; PubMed Central PMCID: PMC9454542. 36078152 PMC9454542

[14] Provansal M, Roche S, Pastore M, Casanova D, Belondrade M, Alais S, et al. Proteomic consequences of expression and pathological conversion of the prion protein in inducible neuroblastoma N2a cells. Prion. 2010;4(4):292–301. Epub 20101027. doi: 10.4161/pri.4.4.13435 ; PubMed Central PMCID: PMC3268962. 20930564 PMC3268962

[15] Arnould H, Baudouin V, Baudry A, Ribeiro LW, Ardila-Osorio H, Pietri M, et al. Loss of prion protein control of glucose metabolism promotes neurodegeneration in model of prion diseases. PLoS Pathog. 2021;17(10):e1009991. Epub 20211005. doi: 10.1371/journal.ppat.1009991 ; PubMed Central PMCID: PMC8519435. 34610054 PMC8519435

[16] Moore RA, Sturdevant DE, Chesebro B, Priola SA. Proteomics analysis of amyloid and nonamyloid prion disease phenotypes reveals both common and divergent mechanisms of neuropathogenesis. J Proteome Res. 2014;13(11):4620–4634. Epub 20140829. doi: 10.1021/pr500329w ; PubMed Central PMCID: PMC4227561. 25140793 PMC4227561

[17] Tewari D, Sah AN, Bawari S, Nabavi SF, Dehpour AR, Shirooie S, et al. Role of nitric oxide in neurodegeneration: Function, regulation, and inhibition. Curr Neuropharmacol. 2021;19(2):114–126. doi: 10.2174/1570159X18666200429001549 ; PubMed Central PMCID: PMC8033982. 32348225 PMC8033982

[18] Spiers JG, Chen HJC, Bourgognon JM, Steinert JR. Dysregulation of stress systems and nitric oxide signaling underlies neuronal dysfunction in Alzheimer’s disease. Free Radic Biol Med. 2019;134:468–483. Epub 20190202. doi: 10.1016/j.freeradbiomed.2019.01.025 . 30716433

[19] Fernández AP, Serrano J, Rodrigo J, Monleón E, Monzón M, Vargas A, et al. Changes in the expression pattern of the nitrergic system of ovine cerebellum affected by scrapie. J Neuropathol Exp Neurol. 2007;66(3):196–207. doi: 10.1097/01.jnen.0000248557.37832.b4 . 17356381

[20] Wang V, Chuang TC, Hsu YD, Chou WY, Kao MC. Nitric oxide induces prion protein via MEK and p38 MAPK signaling. Biochem Biophys Res Commun. 2005;333(1):95–100. doi: 10.1016/j.bbrc.2005.05.091 . 15936714

[21] Ju WK, Park KJ, Choi EK, Kim J, Carp RI, Wisniewski HM, et al. Expression of inducible nitric oxide synthase in the brains of scrapie-infected mice. J Neurovirol. 1998;4(4):445–450. doi: 10.3109/13550289809114544 . 9718137

[22] Kim JI, Ju WK, Choi JH, Choi E, Carp RI, Wisniewski HM, et al. Expression of cytokine genes and increased nuclear factor-kappa B activity in the brains of scrapie-infected mice. Brain Res Mol Brain Res. 1999;73(1–2):17–27. doi: 10.1016/s0169-328x(99)00229-6 . 10581394

[23] Park JH, Kim BH, Park SJ, Jin JK, Jeon YC, Wen GY, et al. Association of endothelial nitric oxide synthase and mitochondrial dysfunction in the hippocampus of scrapie-infected mice. Hippocampus. 2011;21(3):319–333. doi: 10.1002/hipo.20753 . 20082297

[24] Bourgognon JM, Spiers JG, Robinson SW, Scheiblich H, Glynn P, Ortori C, et al. Inhibition of neuroinflammatory nitric oxide signaling suppresses glycation and prevents neuronal dysfunction in mouse prion disease. Proc Natl Acad Sci U S A. 2021;118 (10). doi: 10.1073/pnas.2009579118 ; PubMed Central PMCID: PMC7958397. 33653950 PMC7958397

[25] Bourgognon JM, Spiers JG, Scheiblich H, Antonov A, Bradley SJ, Tobin AB, et al. Alterations in neuronal metabolism contribute to the pathogenesis of prion disease. Cell Death Differ. 2018;25(8):1408–1425. Epub 20180618. doi: 10.1038/s41418-018-0148-x ; PubMed Central PMCID: PMC6113283. 29915278 PMC6113283

[26] Singh N, Das D, Singh A, Mohan ML. Prion protein and metal interaction: physiological and pathological implications. Curr Issues Mol Biol. 2010;12(2):99–107. Epub 20090918. ; PubMed Central PMCID: PMC8284910. 19767653 PMC8284910

[27] Watt NT, Taylor DR, Gillott A, Thomas DA, Perera WS, Hooper NM. Reactive oxygen species-mediated beta-cleavage of the prion protein in the cellular response to oxidative stress. J Biol Chem. 2005;280(43):35914–35921. Epub 20050824. doi: 10.1074/jbc.M507327200 . 16120605

[28] McMahon HE, Mangé A, Nishida N, Créminon C, Casanova D, Lehmann S. Cleavage of the amino terminus of the prion protein by reactive oxygen species. J Biol Chem. 2001;276(3):2286–2291. Epub 20001101. doi: 10.1074/jbc.M007243200 . 11060296

[29] McDonald AJ, Dibble JP, Evans EG, Millhauser GL. A new paradigm for enzymatic control of α-cleavage and β-cleavage of the prion protein. J Biol Chem. 2014;289(2):803–813. Epub 20131118. doi: 10.1074/jbc.M113.502351 ; PubMed Central PMCID: PMC3887206. 24247244 PMC3887206

[30] Brown DR, Wong BS, Hafiz F, Clive C, Haswell SJ, Jones IM. Normal prion protein has an activity like that of superoxide dismutase. Biochem J. 1999;344(Pt 1):1–5. ; PubMed Central PMCID: PMC1220606 10548526 PMC1220606

[31] Stys PK, You H, Zamponi GW. Copper-dependent regulation of NMDA receptors by cellular prion protein: implications for neurodegenerative disorders. J Physiol. 2012;590(6):1357–1368. Epub 20120206. doi: 10.1113/jphysiol.2011.225276 ; PubMed Central PMCID: PMC3382327. 22310309 PMC3382327

[32] Taylor DR, Watt NT, Perera WS, Hooper NM. Assigning functions to distinct regions of the N-terminus of the prion protein that are involved in its copper-stimulated, clathrin-dependent endocytosis. J Cell Sci. 2005;118(Pt 21):5141–5153. doi: 10.1242/jcs.02627 . 16254249

[33] Gasperini L, Meneghetti E, Pastore B, Benetti F, Legname G. Prion protein and copper cooperatively protect neurons by modulating NMDA receptor through S-nitrosylation. Antioxid Redox Signal. 2015;22(9):772–784. Epub 20150204. doi: 10.1089/ars.2014.6032 ; PubMed Central PMCID: PMC4361008. 25490055 PMC4361008

[34] Amin L, Nguyen XT, Rolle IG, D’Este E, Giachin G, Tran TH, et al. Characterization of prion protein function by focal neurite stimulation. J Cell Sci. 2016;129(20):3878–3891. Epub 20160902. doi: 10.1242/jcs.183137 . 27591261

[35] Meneghetti E, Gasperini L, Virgilio T, Moda F, Tagliavini F, Benetti F, et al. Prions strongly reduce NMDA receptor S-nitrosylation levels at pre-symptomatic and terminal stages of prion diseases. Mol Neurobiol. 2019;56(9):6035–6045. Epub 20190201. doi: 10.1007/s12035-019-1505-6 . 30710214

[36] Salzano G, Giachin G, Legname G. Structural consequences of copper binding to the prion protein. Cell. 2019;8(8). Epub 20190725. doi: 10.3390/cells8080770 ; PubMed Central PMCID: PMC6721516. 31349611 PMC6721516

[37] Gasperini L, Meneghetti E, Legname G, Benetti F. In absence of the cellular prion protein, alterations in copper metabolism and copper-dependent oxidase activity affect iron distribution. Front Neurosci. 2016;10:437. Epub 20160927. doi: 10.3389/fnins.2016.00437 ; PubMed Central PMCID: PMC5037227. 27729845 PMC5037227

[38] Mattie MD, McElwee MK, Freedman JH. Mechanism of copper-activated transcription: activation of AP-1, and the JNK/SAPK and p38 signal transduction pathways. J Mol Biol. 2008;383(5):1008–1018. Epub 20080909. doi: 10.1016/j.jmb.2008.08.080 ; PubMed Central PMCID: PMC2662727. 18793645 PMC2662727

[39] Stoytcheva ZR, Vladimirov V, Douet V, Stoychev I, Berry MJ. Metal transcription factor-1 regulation via MREs in the transcribed regions of selenoprotein H and other metal-responsive genes. Biochim Biophys Acta. 2010;1800(3):416–424. Epub 20091112. doi: 10.1016/j.bbagen.2009.11.003 ; PubMed Central PMCID: PMC2826586. 19913599 PMC2826586

[40] Mettert EL, Kiley PJ. Fe-S proteins that regulate gene expression. Biochim Biophys Acta. 2015;1853(6):1284–1293. Epub 20141120. doi: 10.1016/j.bbamcr.2014.11.018 ; PubMed Central PMCID: PMC4390428. 25450978 PMC4390428

[41] Ehsani S, Salehzadeh A, Huo H, Reginold W, Pocanschi CL, Ren H, et al. LIV-1 ZIP ectodomain shedding in prion-infected mice resembles cellular response to transition metal starvation. J Mol Biol. 2012;422(4):556–574. Epub 20120609. doi: 10.1016/j.jmb.2012.06.003 ; PubMed Central PMCID: PMC5006934. 22687393 PMC5006934

[42] Nakic N, Tran TH, Novokmet M, Andreoletti O, Lauc G, Legname G. Site-specific analysis of N-glycans from different sheep prion strains. PLoS Pathog. 2021;17 (2):e1009232. Epub 20210218. doi: 10.1371/journal.ppat.1009232 PubMed Central PMCID: PMC7891774. 33600485 PMC7891774

[43] Korth C, Kaneko K, Prusiner SB. Expression of unglycosylated mutated prion protein facilitates PrP(Sc) formation in neuroblastoma cells infected with different prion strains. J Gen Virol. 2000;81(Pt 10):2555–2563. doi: 10.1099/0022-1317-81-10-2555 . 10993946

[44] Xiao SJ, Hu PP, Xiao GF, Wang Y, Liu Y, Huang CZ. Label-free detection of prion protein with its DNA aptamer through the formation of T-Hg2+−T configuration. J Phys Chem B. 2012;116(32):9565–9569. Epub 20120808. doi: 10.1021/jp302522b . 22823483

[45] Corda E, Du X, Shim SY, Klein AN, Siltberg-Liberles J, Gilch S. Interaction of Peptide Aptamers with Prion Protein Central Domain Promotes alpha-Cleavage of PrP(C). Mol Neurobiol. 2018;55(10):7758–7774. Epub 20180219. doi: 10.1007/s12035-018-0944-9 ; PubMed Central PMCID: PMC6132731. 29460268 PMC6132731

[46] Hay AJD, Murphy TJ, Popichak KA, Zabel MD, Moreno JA. Adipose-derived mesenchymal stromal cells decrease prion-induced glial inflammation in vitro. Sci Rep. 2022;12(1):22567. Epub 20221229. doi: 10.1038/s41598-022-26628-7 ; PubMed Central PMCID: PMC9800558. 36581683 PMC9800558

[47] Bourgognon JM, Steinert JR. The metabolome identity: basis for discovery of biomarkers in neurodegeneration. Neural Regen Res. 2019;14(3):387–390. Epub 2018/12/13. doi: 10.4103/1673-5374.245464 ; PubMed Central PMCID: PMC6334598. 30539802 PMC6334598

